# Genome-Wide Association Study Reveals Additive and Non-Additive Effects on Growth Traits in Duroc Pigs

**DOI:** 10.3390/genes13081454

**Published:** 2022-08-16

**Authors:** Yahui Xue, Shen Liu, Weining Li, Ruihan Mao, Yue Zhuo, Wenkai Xing, Jian Liu, Chuang Wang, Lei Zhou, Minggang Lei, Jianfeng Liu

**Affiliations:** 1 College of Animal Science and Technology, China Agricultural University, Beijing 100193, China; 2 School of Life Science and Engineering, Foshan University, Foshan 528231, China; 3 Jiangxi Zhengbang Breeding Co., Ltd., Nanchang 330096, China; 4 College of Animal Science and Technology, Huazhong Agricultural University, Wuhan 430070, China

**Keywords:** growth traits, Duroc pigs, dominance, epistasis, GWAS, DEBVs

## Abstract

Growth rate plays a critical role in the pig industry and is related to quantitative traits controlled by many genes. Here, we aimed to identify causative mutations and candidate genes responsible for pig growth traits. In this study, 2360 Duroc pigs were used to detect significant additive, dominance, and epistatic effects associated with growth traits. As a result, a total number of 32 significant SNPs for additive or dominance effects were found to be associated with various factors, including adjusted age at a specified weight (AGE), average daily gain (ADG), backfat thickness (BF), and loin muscle depth (LMD). In addition, the detected additive significant SNPs explained 2.49%, 3.02%, 3.18%, and 1.96% of the deregressed estimated breeding value (DEBV) variance for AGE, ADG, BF, and LMD, respectively, while significant dominance SNPs could explain 2.24%, 13.26%, and 4.08% of AGE, BF, and LMD, respectively. Meanwhile, a total of 805 significant epistatic effects SNPs were associated with one of ADG, AGE, and LMD, from which 11 sub-networks were constructed. In total, 46 potential genes involved in muscle development, fat deposition, and regulation of cell growth were considered as candidates for growth traits, including *CD55* and *NRIP1* for AGE and ADG, *TRIP11* and *MIS2* for BF, and *VRTN* and *ZEB2* for LMD, respectively. Generally, in this study, we detected both new and reported variants and potential candidate genes for growth traits of Duroc pigs, which might to be taken into account in future molecular breeding programs to improve the growth performance of pigs.

## 1. Introduction

Pork is a major meat resource for humans, constituting over 31% of all meat consumed worldwide in 2020 (https://www.fao.org/3/cb1993en/cb1993en_meat.pdf, accessed on 20 July 2022). The huge demand for pork has greatly increased the global production of pork. There is a positive correlation between pig growth rate and meat production, which can be directly used to improve meat production in pig breeding [1]. In pig breeding programs, traits such as adjusted age at a specified weight (AGE), average daily gain (ADG), backfat thickness (BF), and loin muscle depth (LMD) are usually used to measure growth rate and carcass performance, as they are direct indicators of productivity [2]. Thus, understanding the genetic mechanisms of these traits might provide valuable information for marker-assisted selection in pigs. Fortunately, all the growth traits mentioned above have moderate to high heritabilities [3], which indicates that an effective breeding method could directly improve these traits.

The economic traits of animals are often controlled by multiple loci with additive, dominance, epistasis, and environmental interaction effects. Dominance refers to a relationship between two alleles or variants of the same gene, whereas epistasis refers to a relationship between alleles of two different genes. Both of them are known as non-additive effects which result in non-linear effects that control variation in phenotypes. Identification of non-additive effects associated with economic traits is an effective approach to understand the genetic architecture that underlies the complex variation of traits [4,5].

With the advance of whole-genome sequencing and high-throughput genotyping technology, genome-wide association study (GWAS), as effective methods for detecting causative mutations associated with economic traits, have been more and more widely used in humans [6] and domestic animals [7,8]. Several GWASs have been performed to search for QTL and candidate genes for growth traits in many species [9,10,11], especially in pigs [2,12,13,14]. However, most studies have focused on additive genetic effects and ignored non-additive effects. Interestingly, significant dominance effects accounted for 6% of the total phenotypic variance in average daily gain, which were detached in a Duroc population, emphasizing the importance of non-additive genetic effects [15]. Meanwhile, it has been suggested that the effects of dominance and/or epistasis should not be ignored in predicting phenotypes [5,16], as these would provide new insights into the genetic architecture of complex economic traits.

Duroc boars, due to their maximized production performance, are commonly used as terminal boars in modern three-way crossbreeding production systems (Duroc × Landrace × Yorkshire, DLY), which fully utilize heterosis from both maternal and paternal sides. This is the most efficient way to boost the genetic improvement of growth traits, and this study aimed to detect additive and non-additive effects on growth traits to find causative variants and potential candidate genes for growth-related traits.

## 2. Materials and Methods

### 2.1. Animals and Phenotype Data

The data used in this study were all from a nucleus breeding farm in the Jiangxi province of China. After data cleaning, a total number of 2360 Duroc pigs born from 2014 to 2021, including 1559 males and 801 females, were used in our study. Pedigrees can be traced back for three generations. Regarding phenotypes, there were 1854 records for AGE and ADG, 1847 records for BF, and 1651 records for LMD, respectively.

ADG and AGE for each individual was calculated with information provided by automated feeding stations. Daily weights were collected from 30 to 100 kg of body weight, and they were used to calculate AGE and ADG. Phenotypes of BF and LMD were measured individually alive at around 100 kg body weight using Aloka 500 V real-time B-mode ultrasound, according to the Chinese national pig performance testing standard. The ultrasound images were taken from the dorsum at a distance of 5 cm from the dorsal midline in the middle of the last third and fourth ribs of each pig. All phenotypes in this study were adjusted to 100 kg using the formulas shown below:

(1) AGE:

(1) $${\mathrm{AGE}}_{100}={\mathrm{AGE}}_{test}+\left(100-wt\right)\times \left(\frac{{\mathrm{AGE}}_{test}-A}{wt}\right)$$

where ${\mathrm{AGE}}_{test}$ represents measured age, wt represents measured weight, and A is the correction coefficient for sire and dam; $A_{sire}=50.775$ and $A_{dam}=46.415$.

(2) BF:

(2) $${\mathrm{BF}}_{100}={\mathrm{BF}}_{test}+\left(100-wt\right)\left(\frac{{\mathrm{BF}}_{test}}{wt-B}\right)$$

where ${\mathrm{BF}}_{test}$ represents measured BF, wt represents measured weight, and B is the correction coefficient for sire and dam; $B_{sire}=-7.277$ and $B_{dam}=-9.440$.

(3) ADG

(3) $${\mathrm{ADG}}_{100}=\frac{100\;\mathrm{kg}}{{\mathrm{AGE}}_{100}}$$

(4) LMD

(4) $${\mathrm{LMD}}_{100}{=\mathrm{LMD}}_{test}\times \frac{C}{C+\left[D\times \left(wt-100\;\mathrm{kg}\right)\right]}$$

where ${\mathrm{LMD}}_{test}$ represents measured LMD, wt represents measured weight, and C and D are the correction coefficients for boars and dam respectively; $C_{sire}=50.52$, $C_{dam}=52.01,$ and D=0.228.

Descriptive information for the phenotypes is presented in Table 1.

### 2.2. Genotype Data and Quality Control

A total number of 2360 Duroc pigs born from 2016 to 2022 were genotyped using Illumina Porcine SNP50 BeadChip (Illumina, San Diego, USA). Data quality control was conducted using the PLINK (version: 1.90, Christopher C Chang, San Jose, USA) software [17]. The genotype data were filtered by the following procedures: (1) individuals with call rate < 0.95, (2) SNP call rate < 0.95, (3) minor allele frequency < 0.01, (4) Hardy–Weinberg equilibrium *p*-value < 10^−6^, and (5) SNPs located in sex chromosomes or unmapped to the reference genome. After the quality control, there were 31,372 high-quality SNPs and 2360 pigs left. Then, missing genotypes were further imputed by Beagle v5.2 software [18].

### 2.3. Statistical Analyses

The estimated breeding values (EBV) for AGE, ADG, BF, and LMD traits were estimated according to average information restricted maximum likelihood using the pedigree-based best linear unbiased prediction (PBLUP) method and the DMUAI module of DMU software [19], as described below:

(5) $$y_{c}=Xb+Za+e$$

where $y_{c}$ was the vector of corrected phenotypic values; X and Z were the incidence matrices of b and a, respectively; b was the vector of fixed effects, including farm, sex, year–season of birth; and e was the vector of residuals with a normal distribution of $e\sim N\left(0,I\sigma_{e}^{2}\right)$, I being an identity matrix and $\sigma_{e}^{2}$ the residual variance. In the PBLUP model, a was the vector of additive genetic effects with a normal distribution of $a\sim N\left(0,A\sigma_{a}^{2}\right)$, $\sigma_{a}^{2}$ being additive genetic variance and A being a pedigree-based additive genetic relationship matrix. Subsequently, the deregressed EBVs (DEBVs) were obtained with the formula $\mathrm{DEBV}=g_{i}/r_{i}^{2}$, $g_{i}$ being the EBV of the ith individual and $r_{i}^{2}$ being the square of estimated accuracies for the ith individual [20], which were calculated using the blupADC [21] R package.

Then, a linear mixed model, including additive and non-additive SNP effects, was generated by GMAT software [22], which can be written as:

(6) $$y_{dEBV}=1_{n}\mu +ZM_{a}a_{a}+ZM_{d}a_{d}+ZM_{aa}a_{aa}+ZM_{ad}a_{ad}+ZM_{dd}a_{dd}+e$$

where $y_{dEBV}$ is the vector of DEBV; $1_{n}$ is a vector of ones; μ is the overall mean; vectors $a_{a}$, $a_{d}$, $a_{aa}$, $a_{ad},$ and $a_{dd}$ are additive, dominance, additive-by-additive (A × A), additive-by-dominance (A × D), and dominance-by-dominance (D × D) SNP effects, respectively; vector e is a collection of residual effects; Z is a design matrix for all the random model effects; and $M_{a}$, $M_{d}$, $M_{aa}$, $M_{ad},$ and $M_{dd}$ are additive, dominance, A × A, A × D, and D × D SNP matrixes, respectively.

Then, Bonferroni correction was implemented to define the significant threshold. To avoid missing the true hints of linkage, the genome-wide significant and suggestive levels were set as *p* = 0.05/N = 1.59 × 10^−6^ and *p* = 1/N = 3.19 × 10^−5^, respectively, where N is the number of analyzed SNPs. The proportion of DEBV variance explained by significant SNPs was calculated according to [23]. The *p*-values of the epistatic effects were Bonferroni-corrected for multiple testing, which resulted in *p* < 1.03 × 10^−9^ as a significance threshold.

### 2.4. SNP–SNP Network and Linkage Disequilibrium (LD) Analysis

Interaction effects between every two SNPs across the whole genome for growth traits were calculated using GMAT (version: 1.01, Chao Ning, Beijing China) software [22]. Then, the SNP–SNP networks with significant epistatic effects for the studied traits were drawn using the epiNet option within epiSNP (version: 4.2, Yang Da, St. Paul, MN, USA) software [24].

The solid spine method in Haploview software (version: 4.2, Mark Daly, Cambridge, UK) [25] was used to predict the independent LD block.

### 2.5. Identification of Candidate Genes

Functional genes within 1 Mbp centered on each significant SNP were annotated as candidate genes. Identified potential genes were searched using the Ensemble Sus scrofa 11.1 reference genome [26]. Pig QTLdb [27] was used to annotate significant SNPs located in previously mapped QTLs in pigs. Additionally, the GeneCards [28] and NCBI [29] databases were used to query gene functions and determine promising candidates.

## 3. Results

### 3.1. Additive Effects

**Figure 1:**
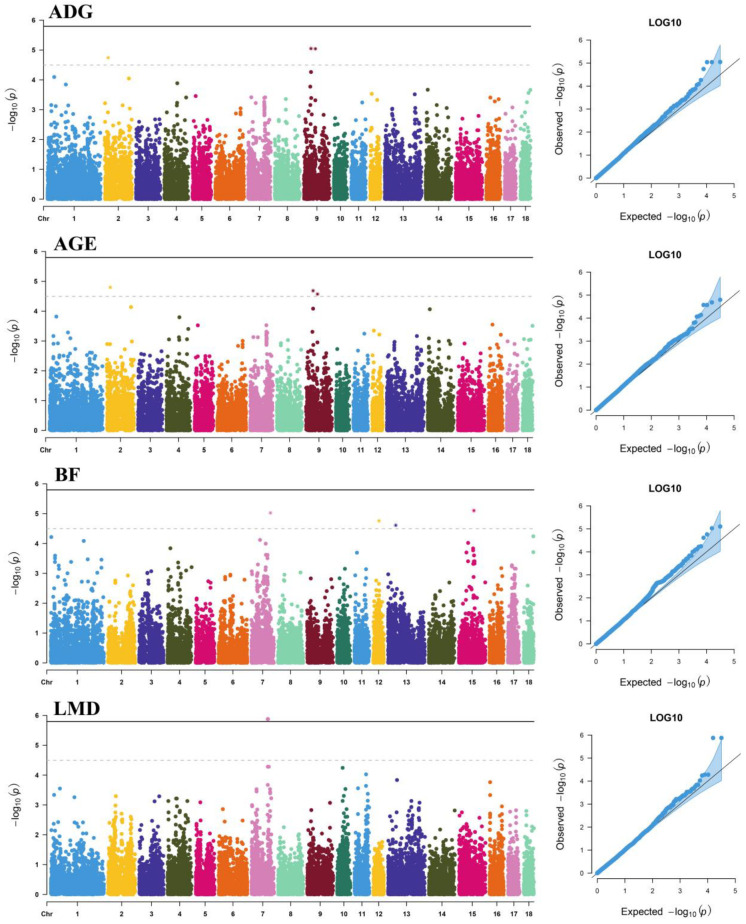
Manhattan plots and *Q*-*Q* plots of SNP additive effects for average daily gain (ADG), adjusted age at 100 kg (AGE), backfat thickness at 100 kg (BF), and loin muscle depth at 100 kg (LMD) traits of Duroc pigs. The *X*-axis shows the physical positions of the SNPs on each chromosome; the *Y*-axis shows the significance levels (−log_10_ *p*-values). The solid line indicates genome-wide significance (*p* < 1.59 × 10^−6^); the dashed line shows suggestive significance (*p* < 3.19 × 10^−5^). The *Q*-*Q* plots show the observed vs. expected log *p*-values.

A total number of 10 SNPs that surpassed the suggestive significance level were detected as being associated with one of the studied traits (Table 2); a Manhattan plot of the −log_10_ (*p*-values) of SNP additive effects is shown in Figure 1. Notice that three SNPs are significantly associated with more than one growth trait, indicating that they exert pleiotropic effects on multiple growth traits. These significant SNPs explained 2.49%, 3.02%, 3.18%, and 1.96% DEBV variance for ADG, AGE, BF, and LMD, respectively. When comparing previously reported QTLs, eight of ten significant SNPs were located in the reported QTL regions (Table S1). The remaining two additive SNPs, CNCB10001620 (Sus scrofa chromosome (SSC) 2: 20310474) and CNCB10006791 (SSC9: 67900715), have not been reported previously in the literature and are likely to be potential novel loci for growth traits in pigs.

### 3.2. Dominance Effects

**Figure 2:**
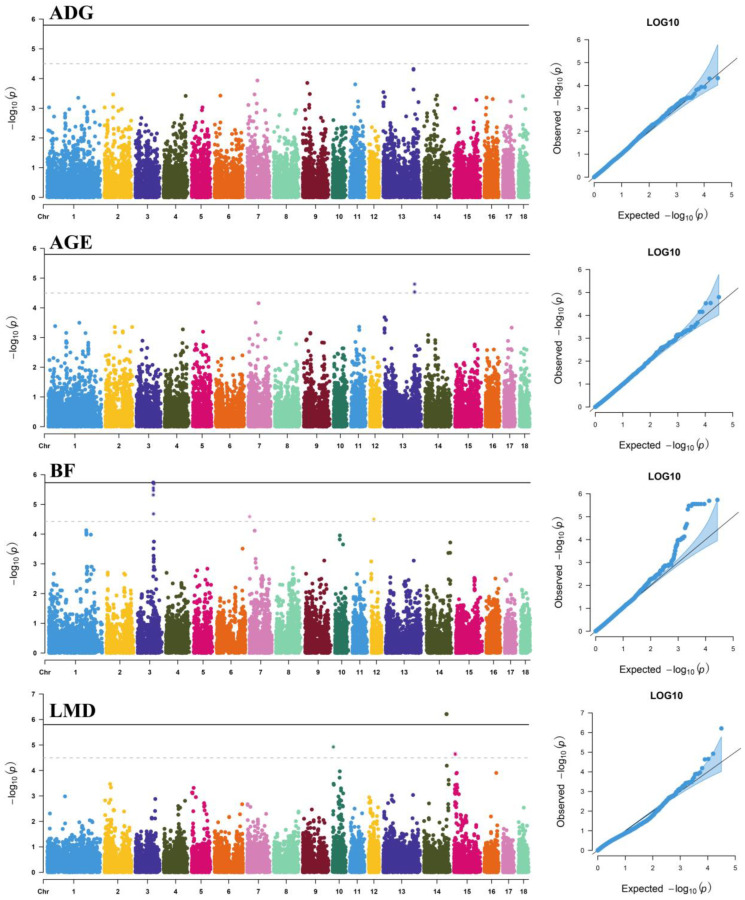
Manhattan plots and *Q*-*Q* plots of SNP dominance effects for average daily gain (ADG), adjusted age at 100 kg (AGE), backfat thickness at 100 kg (BF), and loin muscle depth at 100 kg (LMD) traits. The *X*-axis shows the physical position of SNPs on each chromosome; the *Y*-axis shows the significance levels (−log_10_ *p*-values). The solid line indicates genome-wide significance (*p* < 1.59 × 10^−6^); the dashed line shows suggestive significance (*p* < 3.19 × 10^−5^). The *Q*-*Q* plots show the observed vs. expected log *p*-values.

In summary, we also detected 22 SNPs associated with one of the tested studied traits (Table 3), and the *p*-values of the GWAS results were visualized using Manhattan plots and *Q-Q* plots (Figure 2). These significant SNPs explained 2.24%, 13.26%, and 4.08% DEBV variance for AGE, BF, and LMD, respectively. When comparing reported QTLs, 16 significant SNPs were located in the identified QTL regions (Table S2). The remaining six dominance SNPs have not been reported in pigs and are likely to be potential novel loci for growth traits. Additionally, we detected a haplotype block that spanned 492 kb (Figure S1) in BF containing 11 significant SNPs.

### 3.3. Epistatic Analysis

After cleaning, 287, 355, and 163 pairs of SNPs were significantly associated with ADG, AGE, and LMD, respectively (Table 4), while for BF, no significant SNP pairs were detached. Of these significant SNP–SNP interactions, 43.21–49.08% exhibited an A × A interaction, 32.52–38.03% exhibited an A × D interaction, and 17.18–23.00% exhibited a D × D interaction.

To investigate the complicated mechanism of epistasis on growth traits, SNP–SNP networks were constructed for each trait by EPISNP3 [24]. The epistatic interaction sub-networks that contained more than three nodes are shown in Supplementary Figures S2–S9. There were three, five, and three networks detected for ADG, AGE, and LMD, respectively.

**Figure 3:**
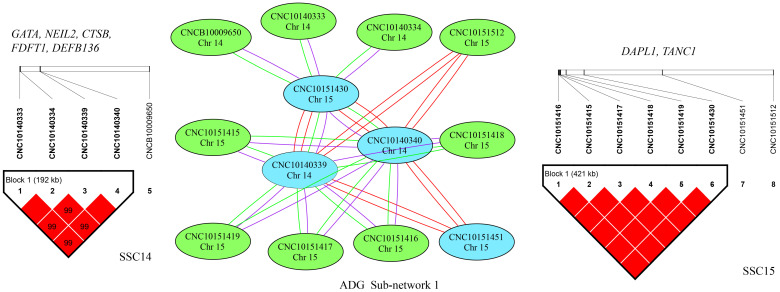
Epistatic sub-network 1 among SNPs affecting ADG and the related LD information. The color of a node represents the *p*-value of an interaction (*p* < 1 × 10^−12^ = red; *p* < 1 × 10^−11^ = blue; *p* < 1 × 10^−10^ = green). The color of connecting lines between circles indicates the type of epistatic effect (A × A = red; A × D = purple; D × D = green). The genes located in the LD block are listed.

For ADG, sub-network 1 was the largest one and contained 28 pairs of SNP–SNP interactions between SSC14 and SSC15, which indicated interactions between the two chromosomes (Figure 3). These interactions detected between SSC14 and SSC15 involved five SNPs on SSC14 and eight SNPs on SSC15, which spanned 192 kb (from 14.93 Mbp to 15.12 Mbp) and 422 kb (from 65.71 Mbp to 66.13 Mbp), respectively. According to gene annotation, we found that there were five genes in the 192 kb region on SSC14 and two genes in the 422 kb region on SSC15. Several SNPs in the same LD block on one chromosome appeared in sub-networks 2, 3, 4, and 5, which interacted with a single SNP on another chromosome. More details can be found in Figures S2–S5.

**Figure 4:**
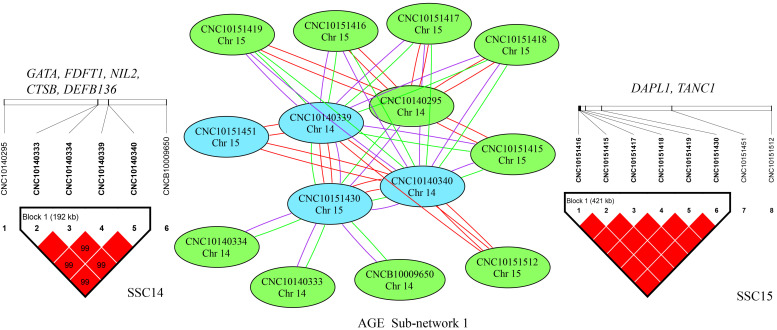
Epistatic sub-network 1 among SNPs affecting AGE and the related LD information. The color of a node represents the *p*-value of an interaction (*p* < 1 × 10^−12^ = red; *p* < 1 × 10^−11^ = blue; *p* < 1 × 10^−10^ = green). The color of the connecting lines between circles indicates the type of epistatic effect (A × A = red; A × D = purple; D × D = green). The genes located in the LD regions are listed.

Interestingly, for AGE, sub-network 1 contained 28 pairs of SNP–SNP interaction effects between SSC14 and SSC15, which was the same as ADG sub-network 1, except for one more SNP on SSC14 (Figure 4). These interactions detected between SSC14 and SSC15 involved five SNPs and eight SNPs respectively, which spanned 192 kb (from 14.93 Mbp to 15.12 Mbp) and 422 kb (from 65.71 Mbp to 66.13 Mbp). There were five genes in the 192 kb region on SSC14 and two genes in the region on SSC15. Several SNPs in the same LD block on one chromosome can be found in sub-networks 2 and 3, which interacted with a single SNP on another chromosome. More details can be found in Figures S6 and S7.

**Figure 5:**
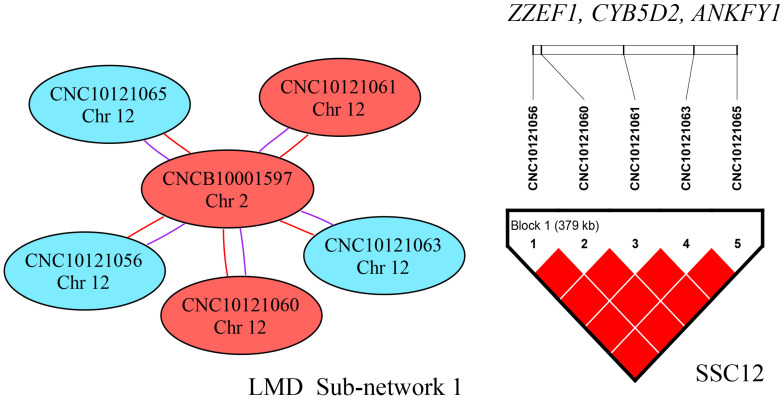
Epistatic sub-network 1 among SNPs affecting LMD and the related LD information. The color of a node represents the *p*-value of an interaction (*p* < 1 × 10^−12^ = red; *p* < 1 × 10^−11^ = blue; *p* < 1 × 10^−10^ = green). The color of connecting lines between circles indicates the type of epistatic effect (A × A = red; A × D = purple; D × D = green). The genes located in the LD regions are listed.

For LMD, we detected 10 significant SNP–SNP interaction effects. These interactions were A × A interactions or A × D interactions, which implicated an interaction between SSC2 and SSC12 (Figure 5). The interactions occurred between five SNPs in a single LD block on SSC12. Multiple SNPs in the same LD block on one chromosome were located in sub-networks 2 and 3, which interacted with a single SNP on another chromosome. More details are shown in Figures S8 and S9.

## 4. Discussion

To eliminate the contribution of information from relatives, which may be significantly associated with the trait analyzed rather than the phenotype, the deregressed EBV (DEBV) was calculated for each individual [30]. DEBV, especially, could take full advantage of the information available on genotyped animals and their relatives, which may properly correct the bias introduced by simply pooling or averaging data information and explaining heterogeneous variance [20]. Therefore, in this study, DEBVs obtained from genetic evaluations were used as response variables to improve the detecting power of GWAS. In recent years, much GWAS research has used DEBVs as pseudo-phenotypes, particularly in work on livestock animals [31,32].

Many significant additive and dominance associations were discovered or involved genomic regions previously reported in the literature. Surprisingly, our study identified a number of novel loci associated with dominance effects on growth traits. SNP–SNP networks of epistatic interactions directly revealed the relationships between SNP effects, which could aid in understanding genetic bias with respect to growth traits.

In this study, the SNP–SNP interactions which occurred on the same chromosome were discovered as interactions between two SNPs on the same chromosome. However, remarkably, these may have been haplotype effects and not interactions [33]. Meanwhile, only interactions involving 10 animals in each genotype combination were considered in order to increase the power of detaching interactions [34]. Then, the results were filtered according to these criteria and large numbers of interactions were identified.

Due to the negative genetic correlation between ADG and AGE, the analysis of these two traits used different modeling approaches to detect candidate genes for growth traits. As a result, some commonly detected signals and some differences were also found as well. There were three significant additive SNPs detected for both ADG and AGE, while, partially overlapped with these, there were SNPs identified as being significantly associated with ADG in Yorkshire and Pietrain pigs [2,35]. The *CD55 molecule* (*CD55*) gene, which plays an important role in adipocyte development and could affect the growth rates of animals by regulating adipocyte production, was identified in nearby candidate SNPs (CNCB10006791 and CNCB10006792) [36,37]. The *nuclear receptor interacting protein 1* (*NRIP1*) gene encodes a nuclear protein also known as *receptor-interacting protein 140* (*RIP140*), which was identified as a candidate gene for AGE with dominant effect. *RIP140* is widely expressed and is involved in regulating lipid and glucose metabolism and fat cell regulation [38,39,40,41]. The *KH RNA binding domain-containing*, *signal transduction-associated 2* (*KHDRBS2*) gene encodes an RNA-binding protein, which is involved in mediating uterine endometrial stroma progenitor development [42]. Studies have shown that *KHDRBS2* is associated with number of teats in Yorkshire pigs [43] and with reproductive traits in Polish commercial pig breeds [44]. Meanwhile, the *farnesyl-diphosphate farnesyltransferase 1* (*FDFT1*) gene was reported as being associated with growth rate through the muscle transcriptome in Iberian pigs [45].

For BF, the *thyroid hormone receptor interactor 11* (*TRIP11*) gene encodes a protein product that interacts with thyroid hormone receptor β, which functions in regulating lipid metabolism [46] and was also found to be associated with intramuscular fat content in a Meishan × Duroc crossbred population [47]. The *musashi homolog 2* (*MIS2*) gene encodes an RNA-binding protein and plays a central role in controlling feed intake and feeding behavior in mammals [48,49]. A haplotype block that spanned 492 kb among significant dominance SNPs was harbored in a reported BF QTL in Duroc, Yorkshire, and Pietrain pigs [50]. Our study increased the power of QTL detection and narrowed the QTL locations using high-density chip arrays and large sample size.

As for LMD, the *vertnin* (*VRTN*) gene is known to affect the variation in vertebral number, and, furthermore, an allele of *VRTN* affects the length of the longissimus muscle, as reported in [51,52]. Interestingly, two significant additive SNPs within or nearby *VRTN* was found in the current study to be associated with LMD. In addition, the *Zinc Finger E-Box Binding Homeobox 2* (*ZEB2*) gene was proved to play a role in skeletal muscle differentiation in pluripotent stem cells [53]. Although it has not been reported in pigs, it would be worth seeking to verify it in the future. Additionally, considering the complexity of implementing a breeding program, the results obtained here may not be sufficient for effective incorporation into programs, though they could be combined with other omics data, such as proteomics and metabolomics data.

## 5. Conclusions

In conclusion, 32 significant SNPs—10 for additive and 22 for dominance effects—and many epistatic interactions were identified for one of four growth traits in a purebred Duroc population through a GWAS fitting additive and non-additive genetic effects. Furthermore, 46 candidate genes with potential functions in muscle development, fat deposition, and regulation of cell growth were considered as candidates for growth traits, including *CD55* and *NRIP1* for AGE and ADG, *TRIP11* and *MIS2* for BF, and *VRTN* and *ZEB2* for LMD, respectively. This study presents novel putative causative variants and genes for future pig breeding programs, which may be used in developing trait-specific marker-assisted selection models.

## Figures and Tables

**Table 1:** Summary statistics for ADG, AGE, BF, and LMD.

Trait	Mean ± SD	Max.	Min.	C.V. (%)	Number of Records
ADG (g/day)	661.66 ± 57.52	824.94	512.03	9.10	1854
AGE (day)	152.33 ± 13.86	195.30	121.22	17.67	1854
BF (mm)	9.58 ± 1.69	15.54	5.70	8.69	1847
LMD (mm)	54.87 ± 6.52	74.01	27.35	11.89	1651

AGE: adjusted age at 100 kg, AGE: average daily gain, BF: backfat thickness, LMD: loin muscle depth, SD: standard deviation, Max.: maximum, Min.: minimum, C.V.: coefficient of variation.

**Table 2:** Summary information of significant additive SNPs for ADG, AGE, BF, and LMD.

Trait	SNP	Chr	Location (bp)	Allele	MAF	*p*-Value	% DEBV	Nearest Gene	Distance (bp)
ADG	CNCB10006667	9	41,538,619	C/T	0.086	8.95 × 10^−6^	0.83	*HTR3B*	Within
	CNCB10006791	9	67,900,715	C/T	0.086	9.09 × 10^−6^	0.83	*CD55*	+32,354
	CNCB10006792	9	67,967,237	A/G	0.086	9.09 × 10^−6^	0.83	*CD55*	−6855
AGE	CNCB10001620	2	20,310,474	A/G	0.216	1.59 × 10^−5^	0.78	*/*	/
	CNCB10006667	9	41,538,619	C/T	0.086	2.07 × 10^−5^	0.76	*HTR3B*	Within
	CNCB10006791	9	67,900,715	C/T	0.086	2.68 × 10^−5^	0.74	*CD55*	+32,354
	CNCB10006792	9	67,967,237	A/G	0.086	2.68 × 10^−5^	0.74	*CD55*	−6855
BF	CNCB10010792	15	89,052,971	G/A	0.051	7.89 × 10^−6^	0.84	*/*	/
	CNCB10005591	7	113,533,476	T/G	0.074	9.44 × 10^−6^	0.82	*TRIP11*	Within
	rs81433919	12	33,673,968	G/A	0.116	1.74 × 10^−5^	0.77	*MSI2*	Within
	CNCB10008592	13	45,621,675	G/A	0.072	2.45 × 10^−5^	0.75	*PRICKLE2*	Within
LMD	rs709317845	7	97,614,602	T/G	0.469	1.33 × 10^−6^	0.98	*VRTN*	+105
	CNC11071978	7	97,615,897	A/G	0.469	1.33 × 10^−6^	0.98	*VRTN*	Within

AGE: adjusted age at 100 kg, AGE: average daily gain, BF: backfat thickness, LMD: loin muscle depth, Chr: chromosome, Location: SNP position in Ensembl, % DEBV: percentage of DEBV variance explained by the SNP, Distance: distance between the nearest gene and the corresponding significant SNP.

**Table 3:** Summary information of significant dominance SNPs for AGE, BF, and LMD.

Trait	SNP	Chr	Location (bp)	Allele	MAF	*p*-Value	% DEBV	Nearest Gene	Distance (bp)
AGE	CNC10133718	13	179,900,741	C/T	0.285	1.60 × 10^−5^	0.78	*NRIP1*	Within
	rs81441574	13	180,051,342	T/C	0.364	2.90 × 10^−5^	0.73	*NRIP1*	−135,116
	rs80867343	13	180,004,272	G/A	0.362	2.98 × 10^−5^	0.73	*NRIP1*	−88,046
BF	rs81373550	3	93,497,234	A/G	0.061	1.85 × 10^−6^	0.95	*TTC7A*	Within
	CNCB10002800	3	97,223,093	T/C	0.149	2.03 × 10^−6^	0.95	*ZFP36L2*	+19,999
	rs81373610	3	93,655,118	A/C	0.060	2.80 × 10^−6^	0.92	*MCFD2*	+22,100
	rs80966590	3	93,722,940	C/T	0.060	2.80 × 10^−6^	0.92	*SOCS5*	−54,462
	rs81475091	3	93,732,314	G/A	0.060	2.80 × 10^−6^	0.92	*SOCS5*	−45,088
	rs81299554	3	93,771,724	A/G	0.060	2.80 × 10^−6^	0.92	*SOCS5*	−5678
	rs81373642	3	93,828,846	C/T	0.060	2.80 × 10^−6^	0.92	*SOCS5*	Within
	rs81373648	3	93,902,023	G/T	0.060	2.80 × 10^−6^	0.92	*SOCS5*	Within
	rs81373727	3	94,084,767	A/G	0.060	3.36 × 10^−6^	0.91	*TMEM247*	+17,743
	rs81373716	3	94,110,331	G/A	0.060	3.36 × 10^−6^	0.91	*TMEM247*	+43,307
	rs81373744	3	94,163,788	G/A	0.060	3.36 × 10^−6^	0.91	*EPAS1*	−3971
	rs81213041	3	93,394,317	G/A	0.059	4.80 × 10^−6^	0.88	*STPG4*	Within
	rs81373880	3	94,599,697	C/T	0.075	2.09 × 10^−5^	0.76	*PRKCE*	Within
	CNC10070016	7	834,427	A/C	0.430	2.57 × 10^−5^	0.74	*GMDS*	Within
	CNC10120338	12	16,082,351	T/C	0.488	3.13 × 10^−5^	0.73	*MRC2*	Within
LMD	rs80785395	14	133,966,414	A/G	0.087	6.14 × 10^−7^	1.04	*LHPP*	Within
	CNCB10007305	10	4,667,593	A/G	0.094	1.19 × 10^−5^	0.80	*/*	/
	rs81304718	15	7,424,079	C/T	0.091	2.21 × 10^−5^	0.76	*ZEB2*	−74,800
	CNCB10010367	15	8,203,810	A/G	0.060	2.34 × 10^−5^	0.75	*ARHGAP15*	Within

AGE: adjusted age at 100 kg, AGE: average daily gain, BF: backfat thickness, LMD: loin muscle depth, Chr: chromosome, Location: SNP position in Ensembl, % DEBV: percentage of DEBV variance explained by the SNP, Distance: distance between the nearest gene and the corresponding significant SNP.

**Table 4:** Summary information of significant epistatic effects for growth traits.

Traits	N	A × A	A × D	D × D
ADG	287	124	97	66
AGE	355	159	135	61
LMD	163	80	53	30

AGE: adjusted age at 100 kg, AGE: average daily gain, LMD: loin muscle depth, N: number of significant SNP pairs, A × A: number of additive-by-additive interactions, A × D: number of additive-by-dominance interactions, D × D: number of dominance-by-dominance interactions.

## Data Availability

The datasets generated and/or analyzed in the current study are not publicly available, since the studied population consists of the nucleus herd of Jiangxi Zhengbang Breeding Co., Ltd., but are available from the corresponding author upon reasonable request.

## Supplementary Materials

The following supporting information can be downloaded at: https://www.mdpi.com/article/10.3390/genes13081454/s1, Table S1: The additive QTL regions detected in the current study overlapped with previous studies; Table S2: The dominance QTL regions detected in the current study overlapped with previous studies; Figure S1: Linkage disequilibrium (LD) blocks in the significant region on SSC3 for BF; Figure S2: Sub-network 2 for ADG and the LD information; Figure S3: Sub-network 3 for ADG and the LD information; Figure S4: Sub-network 4 for ADG and the LD information; Figure S5: Sub-network 5 for ADG and the LD information; Figure S6: Sub-network 2 for AGE and the LD information; Figure S7: Sub-network 3 for AGE and the LD information; Figure S8: Sub-network 2 for LMD and the LD information; Figure S9: Sub-network 3 for LMD and the LD information.
