# Supplementary material for: Genome-Wide Association Study Reveals Additive and Non-Additive Effects on Growth Traits in Duroc Pigs
Source: Genes (Basel). 2022 Aug 16;13(8):1454. doi: 10.3390/genes13081454 (PMC9407794; doi:10.3390/genes13081454)
Supplement: Supplementary file 1 [file genes-13-01454-s001.zip › Supplementary Table S1.pdf]

Table S1 The additive QTL regions detected in the current study overlapped with previous studies.

traits	SSC	SNP ID	QTL			
			ID	QTL Span (Mbp)	traits	reference
AGE & ADG	9	CNCB10006667	170909	41.5-41.5	Average daily gain	[1]
	9	CNCB10006792	130568	69.1-69.1	Average daily gain	[2]
BF	15	CNCB10010792	31284	87.8-87.8	Backfat between 3rd and 4th last ribs	[3]
	7	CNCB10005591	169095	113.6-113.6	Intramuscular fat content	[4]
	12	CNC10120707	65372	33.3-33.3	Intramuscular fat content	[5]
	13	CNCB10008592	7404	17.6-17.7	Intramuscular fat content	[6]
LMD	7	CNC11071977	172691	97.6-97.6	Loin muscle area	[7]
	7	CNC11071978	172692	97.6-97.6	Loin muscle depth	[7]

Table S2 The dominance QTL regions detected in the current study overlapped with previous studies

traits	SSC	SNP ID	QTL			
			ID	QTL Span (Mbp)	traits	reference
BF	3	CNC10031979<br>CNC10031980<br>CNC10031983<br>CNC10031984<br>CNC10031986<br>CNC10031987<br>CNC10031988<br>CNC10031994<br>CNC10031993<br>CNC10031991<br>CNC10031977<br>CNC10032002	31220	94.2-94.2	Backfat between 3rd and 4th last ribs	[3]
	3	CNCB10002800	7530	122.3-126.9	Average backfat thickness	[8]
	7	CNC10070016	193582	0.9-1.5	Average backfat thickness	[9]
LMD	14	CNC10142793	16363	135.0-135.0	Loin muscle area	[10]
	10	CNCB10007305	2999	6.4-11.1	Loin muscle area	[11]

Reference

1. Tang, Z.; Xu, J.; Yin, L.; Yin, D.; Zhu, M.; Yu, M.; Li, X.; Zhao, S.; Liu, X. Genome-wide association study reveals candidate genes for growth relevant traits in pigs. *Front. Genet.* **2019**, *10*, 302, doi:10.3389/fgene.2019.00302.
2. Meng, Q.; Wang, K.; Liu, X.; Zhou, H.; Xu, L.; Wang, Z.; Fang, M. Identification of growth trait related genes in a Yorkshire purebred pig population by genome-wide association studies. *Asian-Australas. J. Anim. Sci.* **2017**, *30*, 462-469, doi:10.5713/ajas.16.0548.
3. Fowler, K.E.; Pong-Wong, R.; Bauer, J.; Clemente, E.J.; Reitter, C.P.; Affara, N.A.; Waite, S.; Walling, G.A.; Griffin, D.K. Genome wide analysis reveals single nucleotide polymorphisms associated with fatness and putative novel copy number variants in three pig breeds. *BMC Genomics* **2013**, *14*, 784, doi:10.1186/1471-2164-14-784.
4. Duarte, D.A.S.; Fortes, M.R.S.; Duarte, M.d.S.; Guimarães, S.E.F.; Verardo, L.L.; Veroneze, R.; Ribeiro, A.M.F.; Lopes, P.S.; de Resende, M.D.V.; Fonseca e Silva, F. Genome-wide association studies, meta-analyses and derived gene network for meat quality and carcass traits in pigs. *Animal Production Science* **2018**, *58*, 1100-1108, doi:10.1071/AN16018.
5. Davoli, R.; Luise, D.; Mingazzini, V.; Zambonelli, P.; Braglia, S.; Serra, A.; Russo, V. Genome-wide study on intramuscular fat in Italian Large White pig breed using the PorcineSNP60 BeadChip. *J. Anim. Breed. Genet.* **2016**, *133*, 277-282, doi:10.1111/jbg.12189.
6. Sanchez, M.P.; Iannuccelli, N.; Basso, B.; Bidanel, J.P.; Billon, Y.; Gandemer, G.; Gilbert, H.; Larzul, C.; Legault, C.; Riquet, J.; et al. Identification of QTL with effects on intramuscular fat content and fatty acid composition in a Duroc x Large White cross. *BMC Genet.* **2007**, *8*, 55, doi:10.1186/1471-2156-8-55.
7. Zhuang, Z.; Li, S.; Ding, R.; Yang, M.; Zheng, E.; Yang, H.; Gu, T.; Xu, Z.; Cai, G.; Wu, Z.; et al. Meta-analysis of genome-wide association studies for loin muscle area and loin muscle depth in two Duroc pig populations. *PLoS ONE* **2019**, *14*, e0218263, doi:10.1371/journal.pone.0218263.
8. Tribout, T.; Iannuccelli, N.; Druet, T.; Gilbert, H.; Riquet, J.; Gueblez, R.; Mercat, M.J.; Bidanel, J.P.; Milan, D.; Le Roy, P. Detection of quantitative trait loci for reproduction and production traits in Large White and French Landrace pig populations. *Genet Sel Evol* **2008**, *40*, 61-78, doi:10.1186/1297-9686-40-1-61.
9. Bergamaschi, M.; Maltecca, C.; Fix, J.; Schwab, C.; Tiezzi, F. Genome-wide association study for carcass quality traits and growth in purebred and crossbred pigs1. *J. Anim. Sci.* **2020**, *98*, doi:10.1093/jas/skz360.
10. Fan, B.; Onteru, S.K.; Du, Z.Q.; Garrick, D.J.; Stalder, K.J.; Rothschild, M.F. Genome-wide association study identifies Loci for body composition and structural soundness traits in pigs. *PLoS ONE* **2011**, *6*, e14726, doi:10.1371/journal.pone.0014726.
11. Rohrer, G.A.; Thallman, R.M.; Shackelford, S.; Wheeler, T.; Koohmaraie, M. A

genome scan for loci affecting pork quality in a Duroc-Landrace F population. *Anim. Genet.* **2006**, *37*, 17-27, doi:10.1111/j.1365-2052.2005.01368.x.

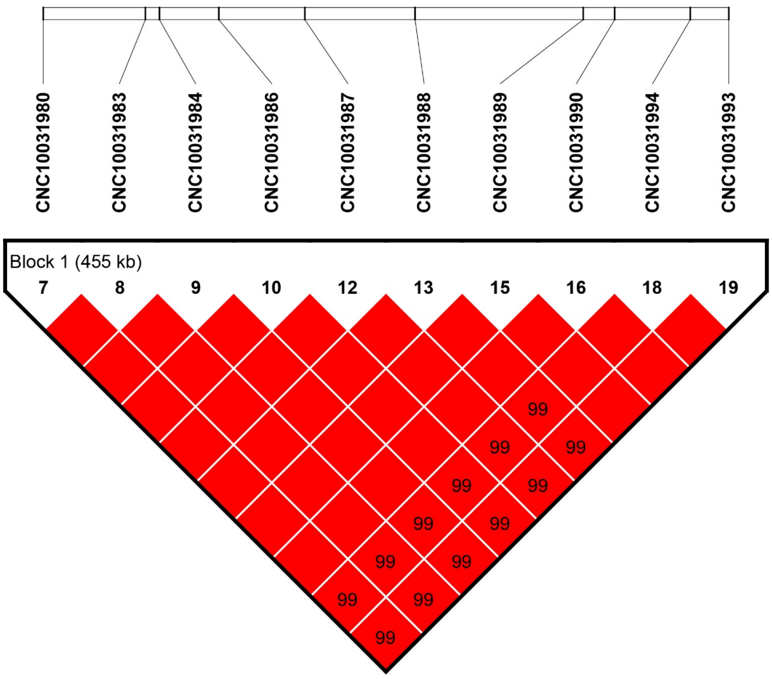

Figure S1. Linkage disequilibrium (LD) blocks in the significant region on SSC3 for BF

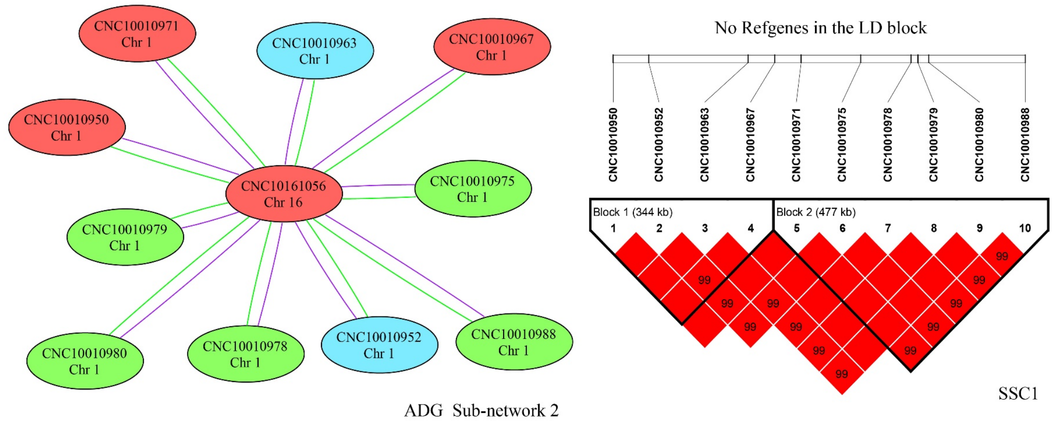

Figure S2. Epistatic sub-network 2 among SNPs affecting ADG and the related LD information. The color of the node represents the *P*-value of an interaction ($P < 1 \times 10^{-12}$ = red; $P < 1 \times 10^{-11}$ = blue; $P < 1 \times 10^{-10}$ = green). The color of the edge indicates the type of epistatic effect (A × A = red; A × D = purple; D × D = green). The genes located in the LD regions were listed.

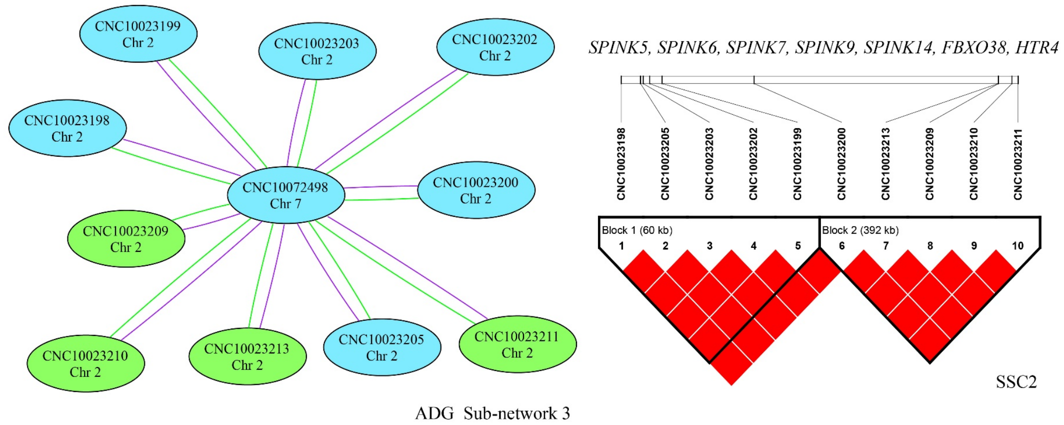

Figure S3. Epistatic sub-network 3 among SNPs affecting ADG and the related LD information. The color of the node represents the *P*-value of an interaction ($P < 1 \times 10^{-12}$ = red; $P < 1 \times 10^{-11}$ = blue; $P < 1 \times 10^{-10}$ = green). The color of the edge indicates the type of epistatic effect (A × A = red; A × D = purple; D × D = green). The genes located in the LD regions were listed.

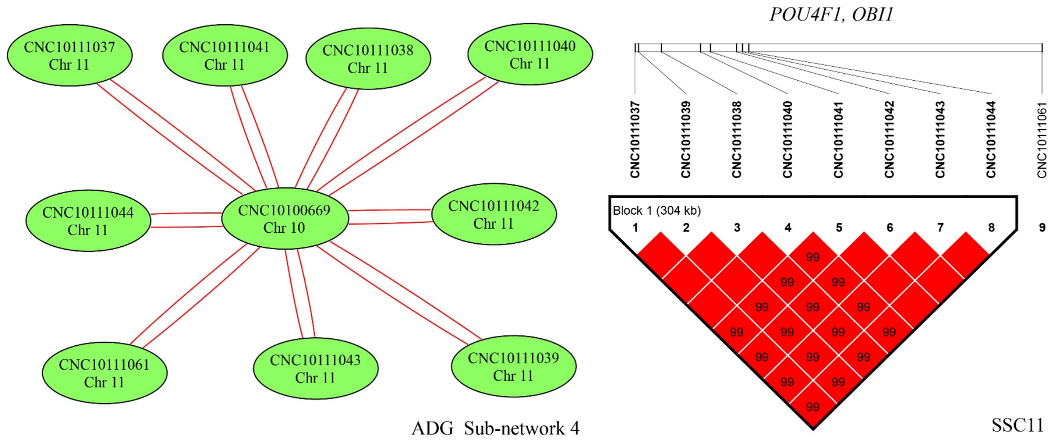

Figure S4. Epistatic sub-network 4 among SNPs affecting ADG and the related LD information. The color of the node represents the *P*-value of an interaction ($P < 1 \times 10^{-12}$ = red; $P < 1 \times 10^{-11}$ = blue; $P < 1 \times 10^{-10}$ = green). The color of the edge indicates the type of epistatic effect (A × A = red; A × D = purple; D × D = green). The genes located in the LD regions were listed.

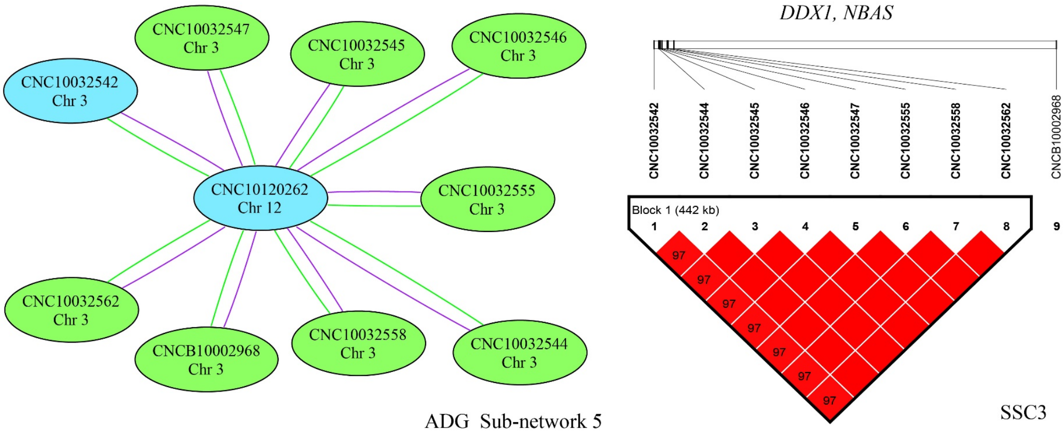

ADG Sub-network 5

Figure S5. Epistatic sub-network 5 among SNPs affecting ADG and the related LD information. The color of the node represents the *P*-value of an interaction ($P < 1 \times 10^{-12}$ = red; $P < 1 \times 10^{-11}$ = blue; $P < 1 \times 10^{-10}$ = green). The color of the edge indicates the type of epistatic effect (A × A = red; A × D = purple; D × D = green). The genes located in the LD regions were listed.

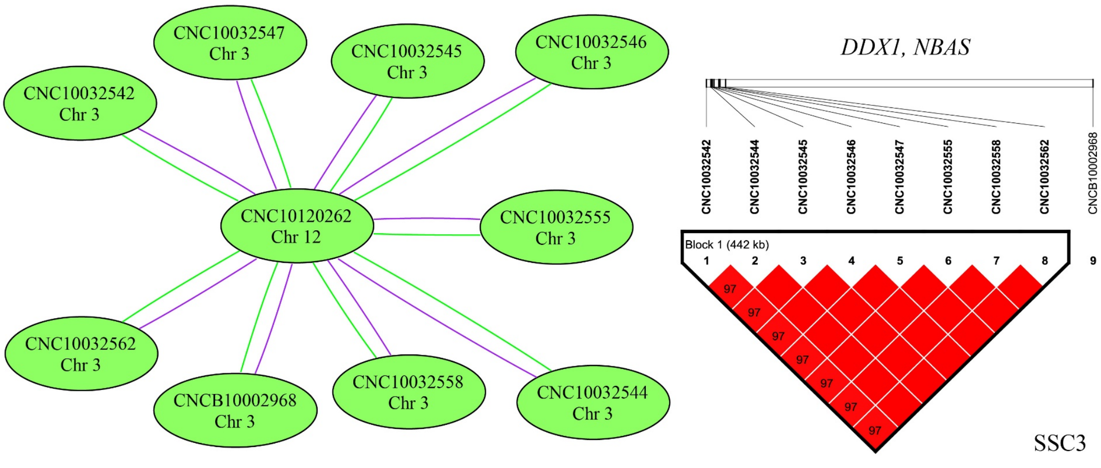

AGE Sub-network 2

Figure S6. Epistatic sub-network 2 among SNPs affecting AGE and the related LD information. The color of the node represents the *P*-value of an interaction ($P < 1 \times 10^{-12}$ = red; $P < 1 \times 10^{-11}$ = blue; $P < 1 \times 10^{-10}$ = green). The color of the edge indicates the type of epistatic effect (A × A = red; A × D = purple; D × D = green). The genes located in the LD regions were listed.

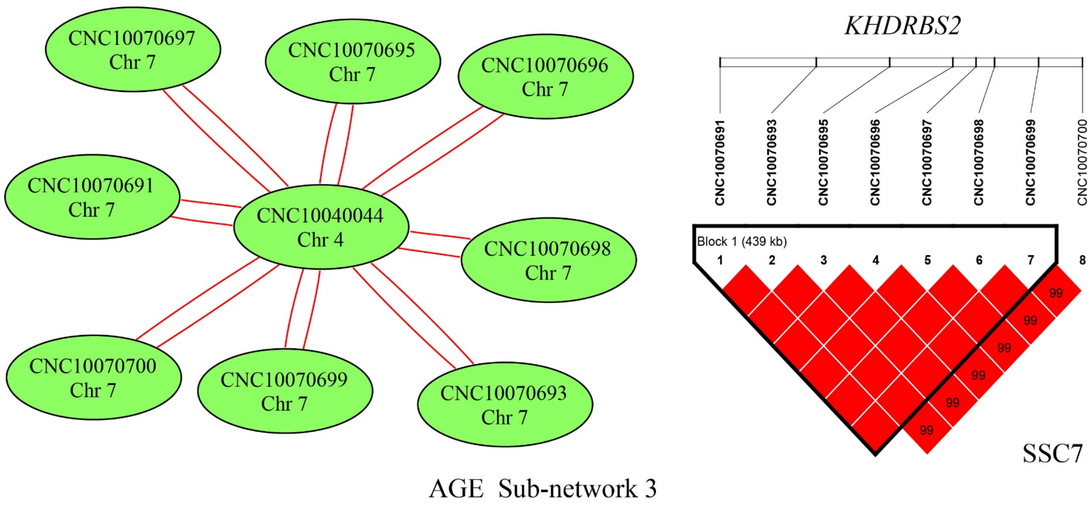

Figure S7. Epistatic sub-network 3 among SNPs affecting AGE and the related LD information. The color of the node represents the *P*-value of an interaction ($P < 1 \times 10^{-12}$ = red; $P < 1 \times 10^{-11}$ = blue; $P < 1 \times 10^{-10}$ = green). The color of the edge indicates the type of epistatic effect (A × A = red; A × D = purple; D × D = green). The genes located in the LD regions were listed.

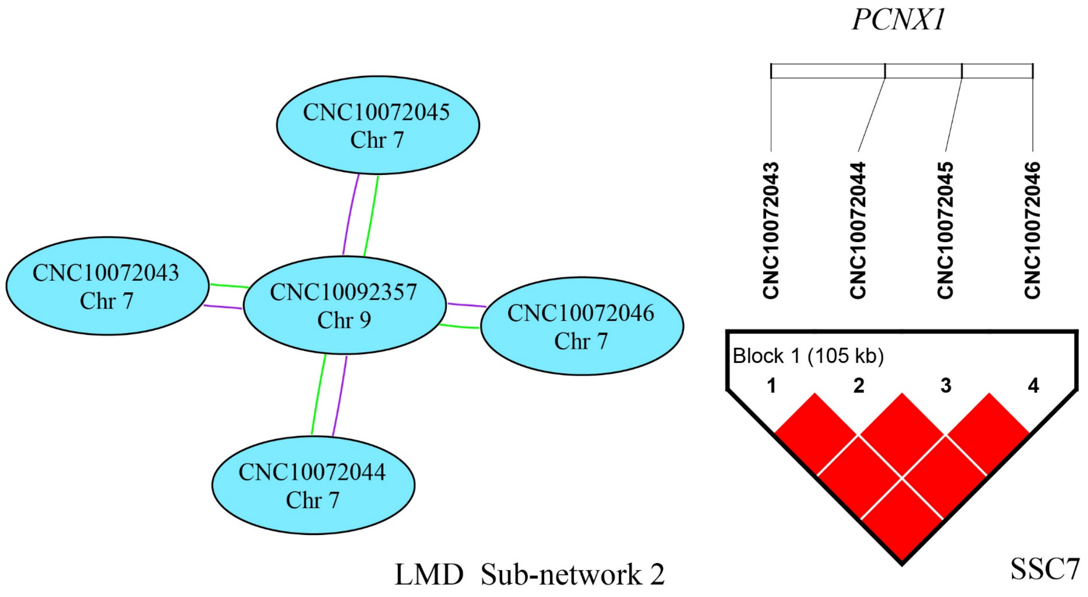

Figure S8. Epistatic sub-network 2 among SNPs affecting LMD and the related LD information. The color of the node represents the *P*-value of an interaction ($P < 1 \times 10^{-12}$ = red; $P < 1 \times 10^{-11}$ = blue; $P < 1 \times 10^{-10}$ = green). The color of the edge indicates the type of epistatic effect (A × A = red; A × D = purple; D × D = green). The genes located in the LD regions were listed.

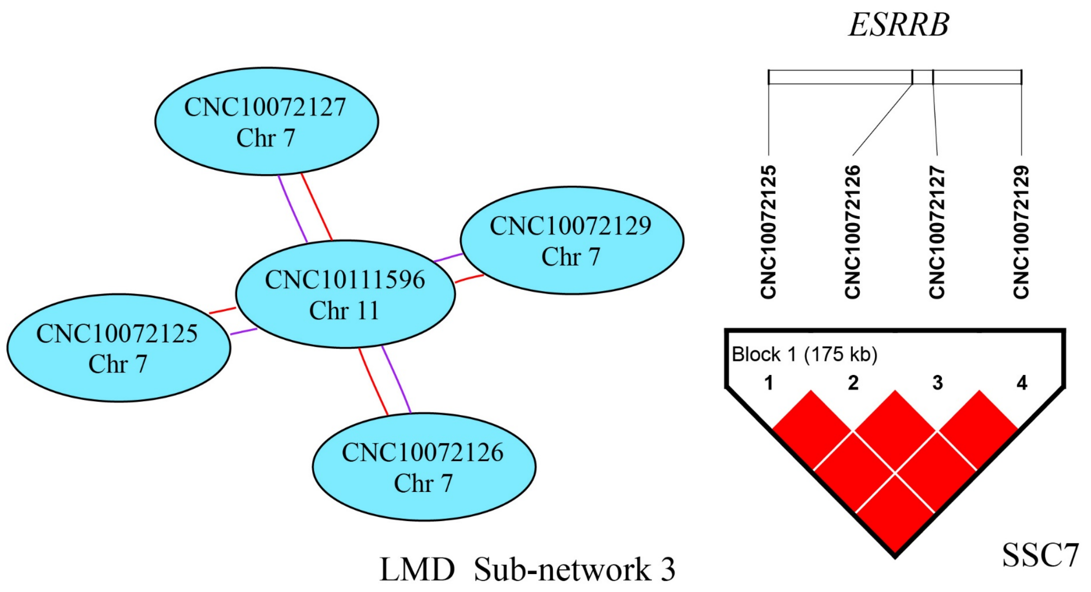

Figure S9. Epistatic sub-network 3 among SNPs affecting LMD and the related LD information. The color of the node represents the *P*-value of an interaction ($P < 1 \times 10^{-12}$ = red; $P < 1 \times 10^{-11}$ = blue; $P < 1 \times 10^{-10}$ = green). The color of the edge indicates the type of epistatic effect (A × A = red; A × D = purple; D × D = green). The genes located in the LD regions were listed.
