# Supplementary figures and images for: Genome-Wide Association Study Reveals Additive and Non-Additive Effects on Growth Traits in Duroc Pigs
Source: Genes (Basel). 2022 Aug 16;13(8):1454. doi: 10.3390/genes13081454 (PMC9407794; doi:10.3390/genes13081454)

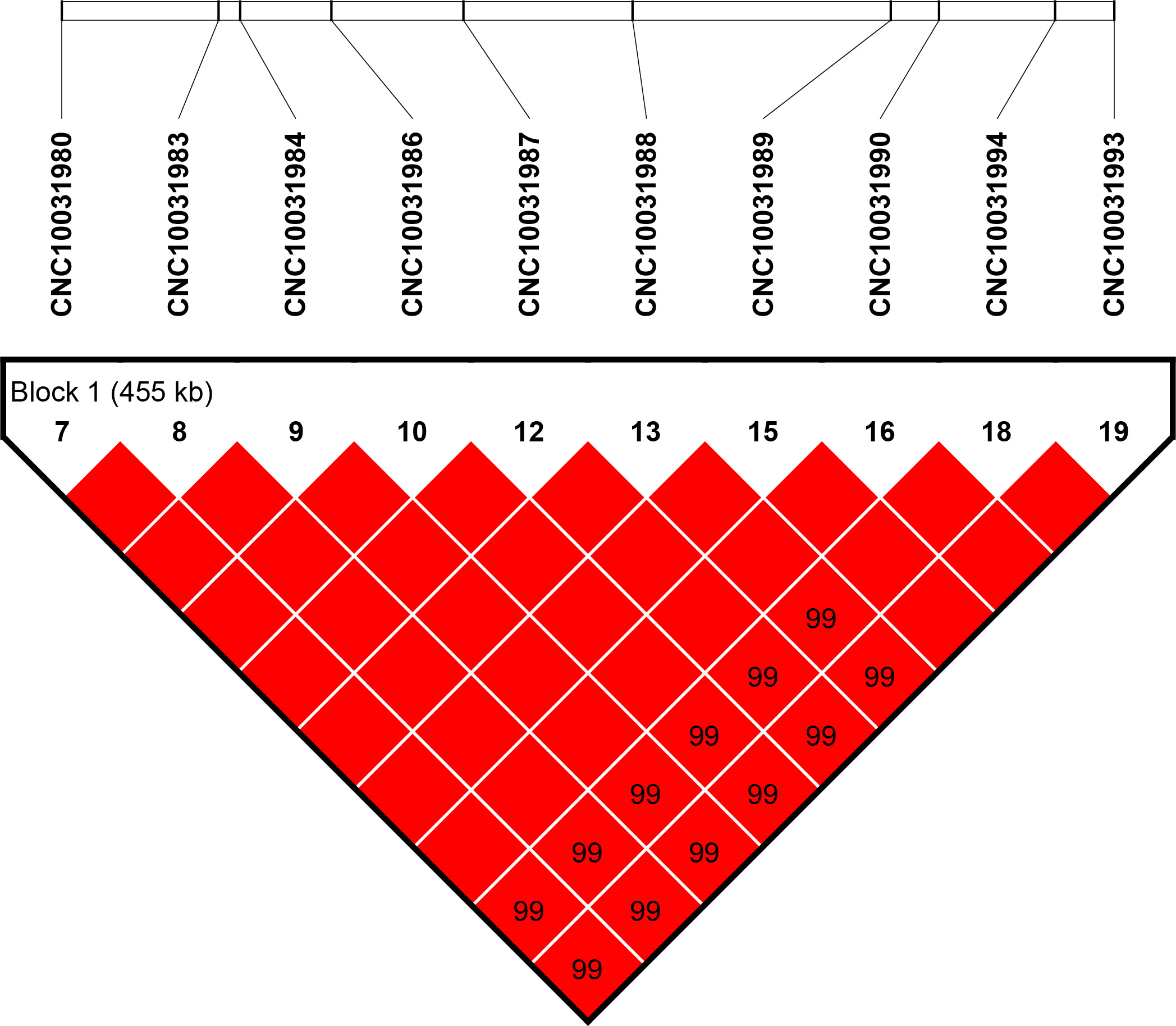

Supplement: Supplementary file 1 [file genes-13-01454-s001.zip › Figure S1. Linkage disequilibrium (LD) blocks in the significant region on SSC3 for BF.jpg]

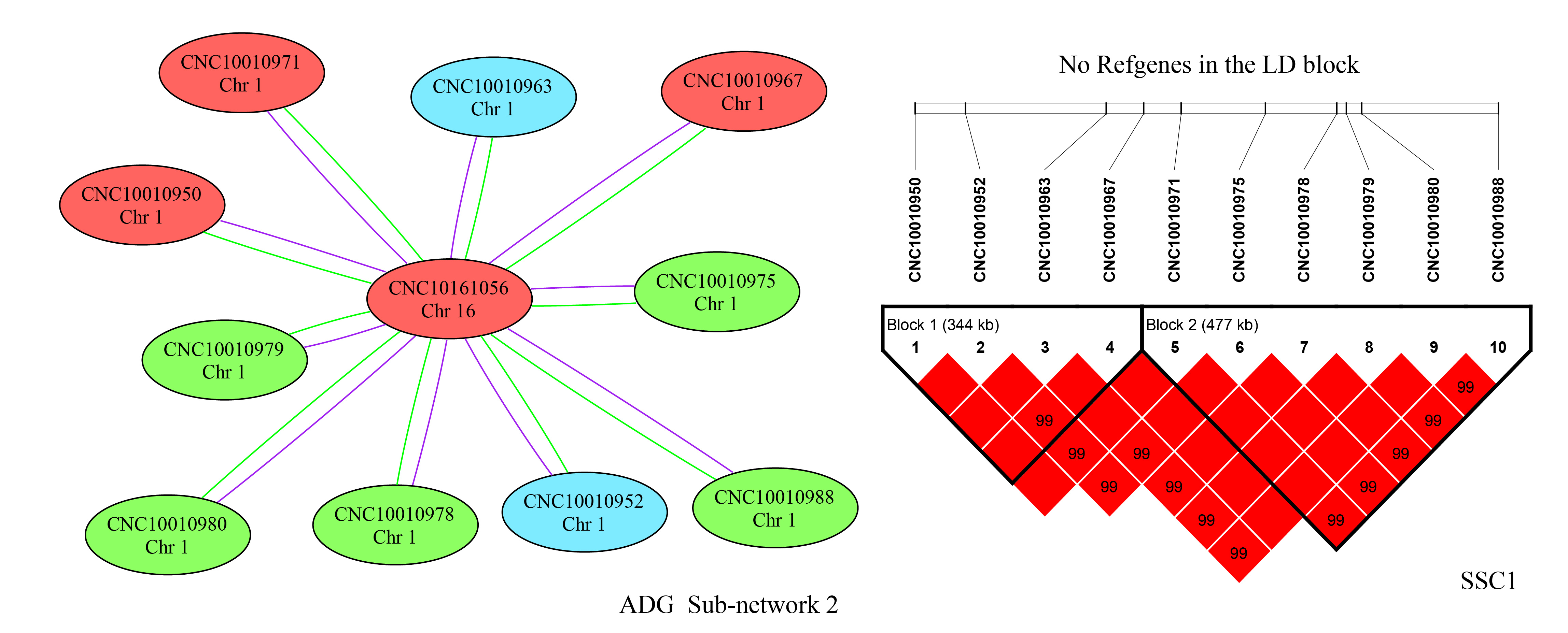

Supplement: Supplementary file 1 [file genes-13-01454-s001.zip › Figure S2. Epistatic sub-network 2 among SNPs affecting ADG and the related LD information.jpg]

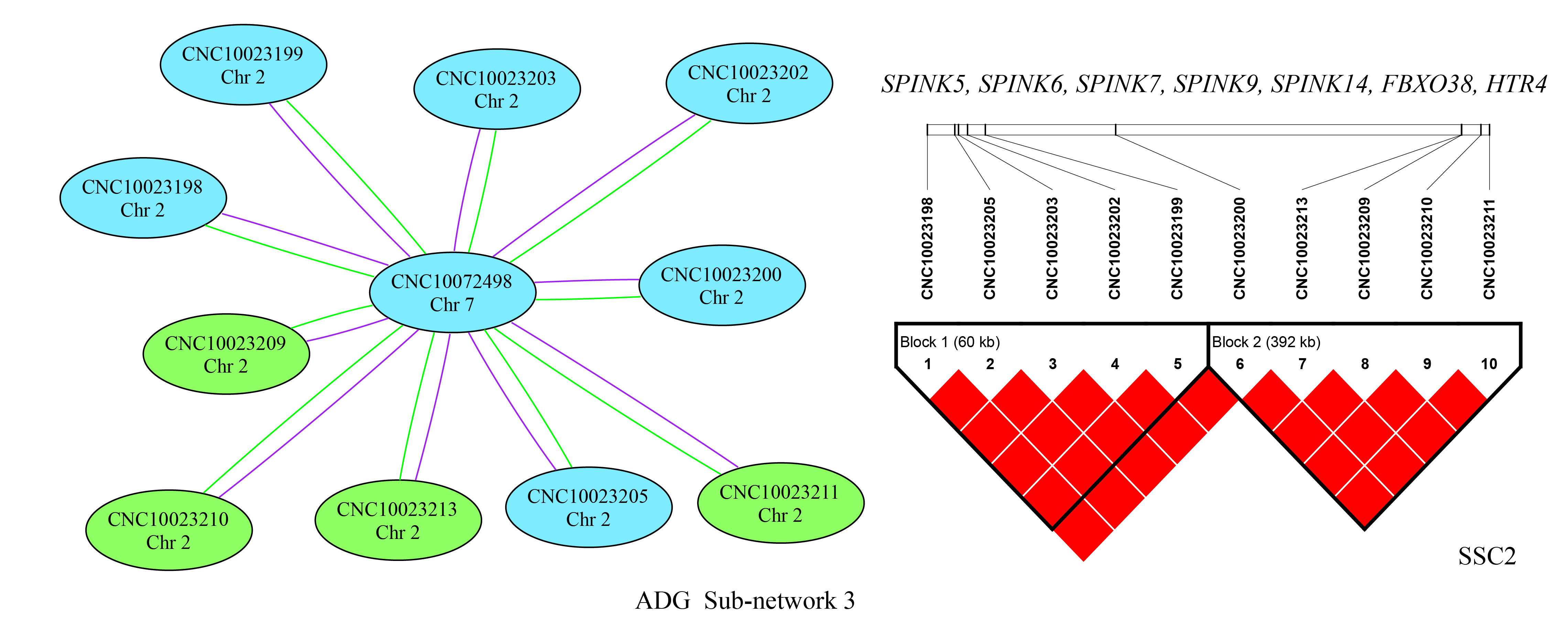

Supplement: Supplementary file 1 [file genes-13-01454-s001.zip › Figure S3. Epistatic sub-network 3 among SNPs affecting ADG and the related LD information.jpg]

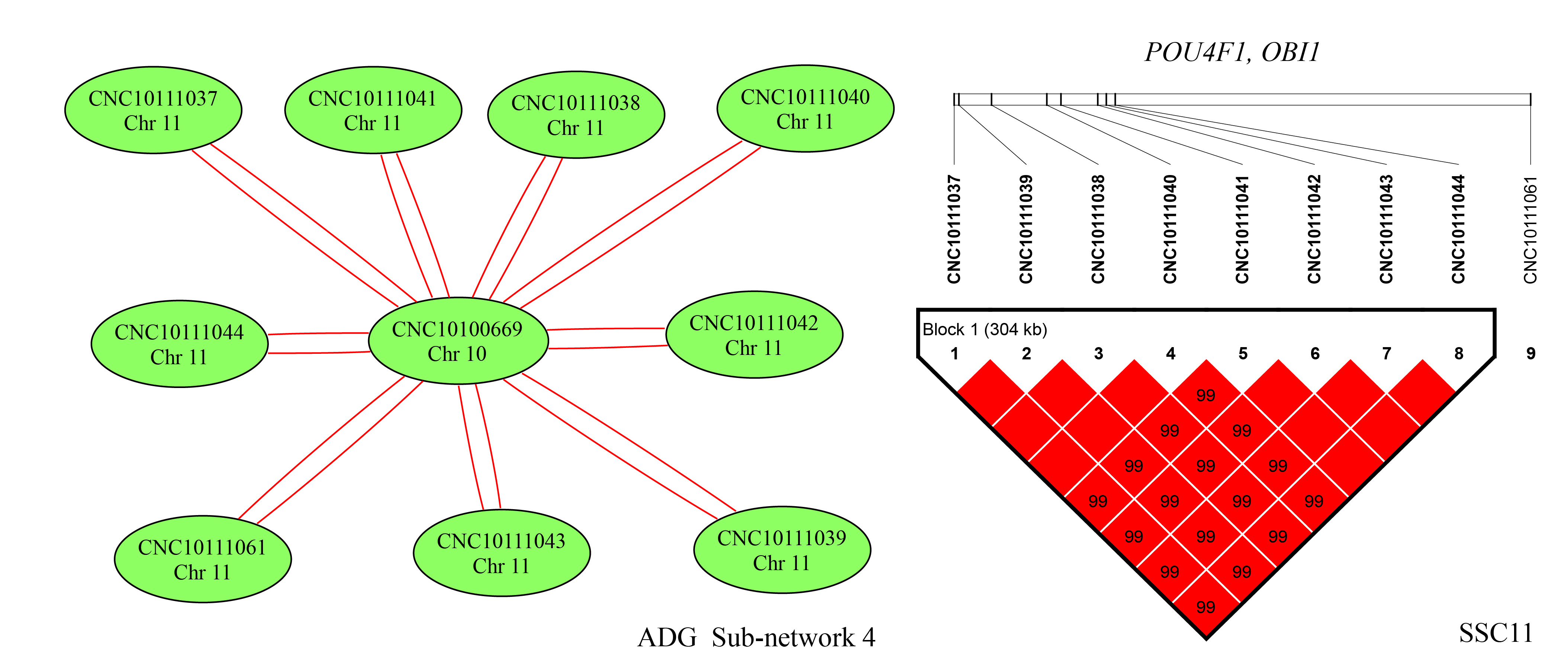

Supplement: Supplementary file 1 [file genes-13-01454-s001.zip › Figure S4. Epistatic sub-network 4 among SNPs affecting ADG and the related LD information.jpg]

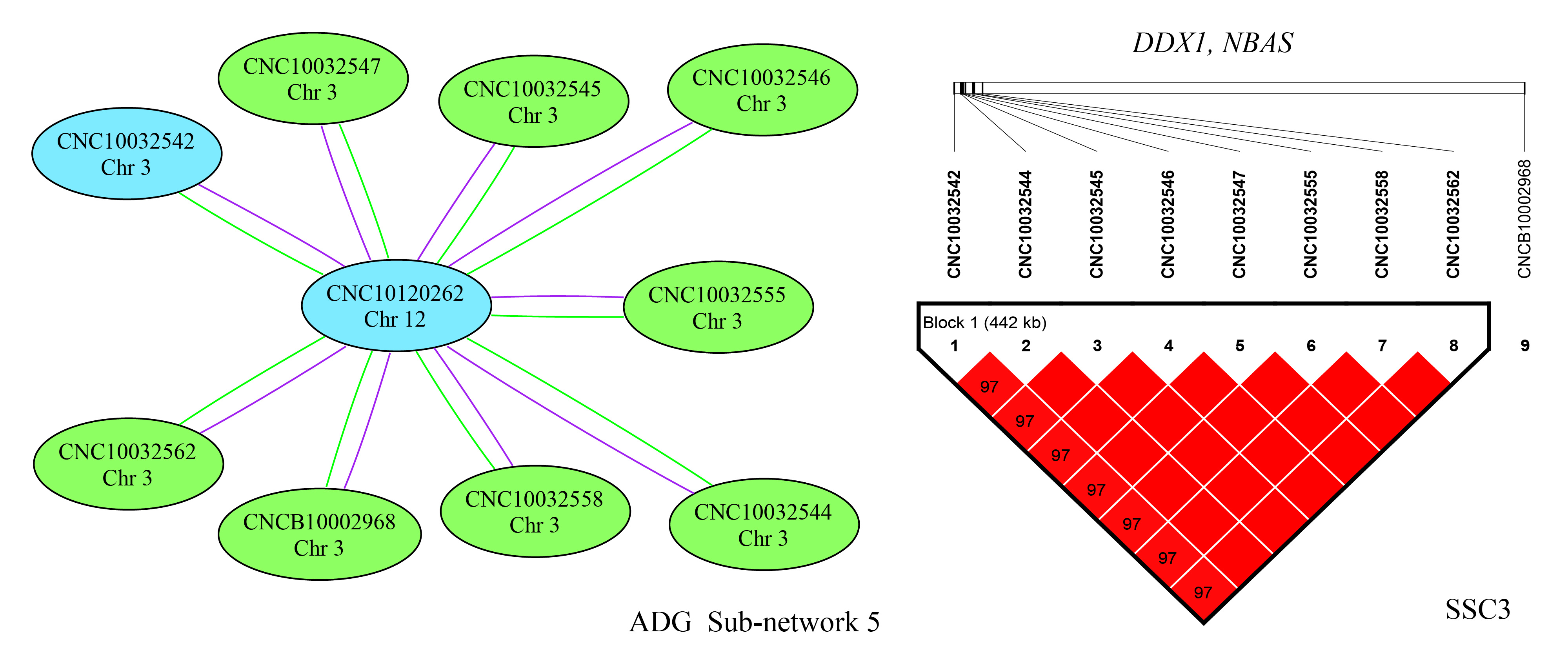

Supplement: Supplementary file 1 [file genes-13-01454-s001.zip › Figure S5. Epistatic sub-network 5 among SNPs affecting ADG and the related LD information.jpg]

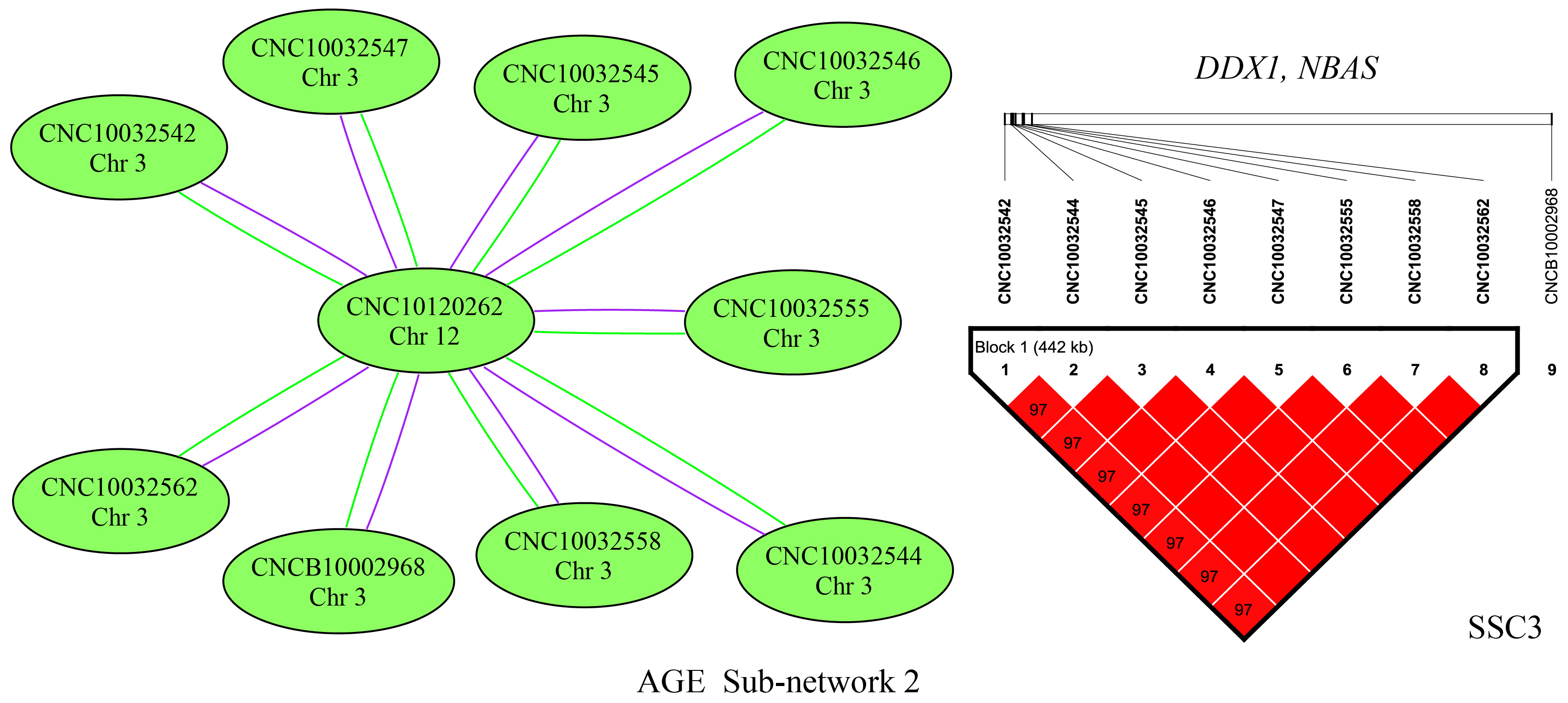

Supplement: Supplementary file 1 [file genes-13-01454-s001.zip › Figure S6. Epistatic sub-network 2 among SNPs affecting AGE and the related LD information.jpg]

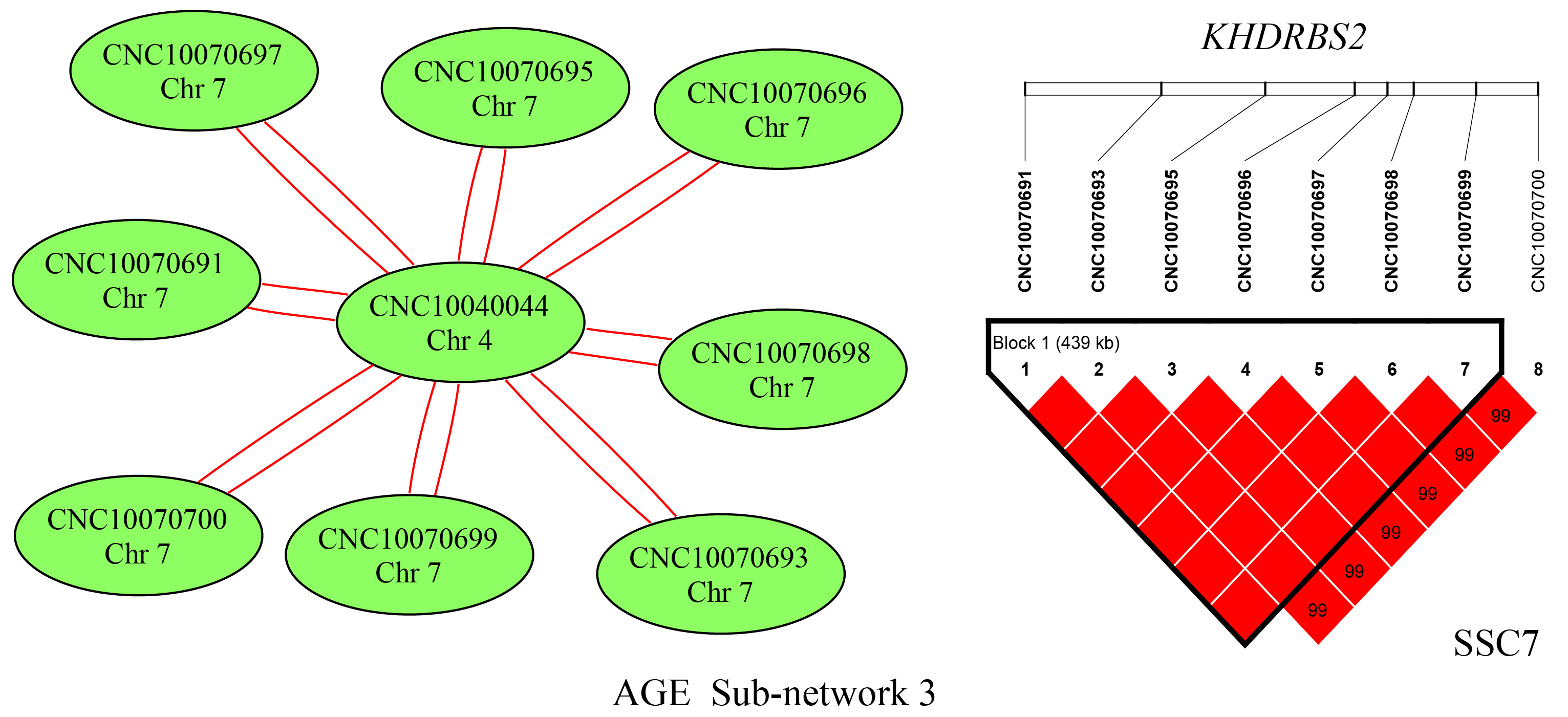

Supplement: Supplementary file 1 [file genes-13-01454-s001.zip › Figure S7. Epistatic sub-network 3 among SNPs affecting AGE and the related LD information.jpg]

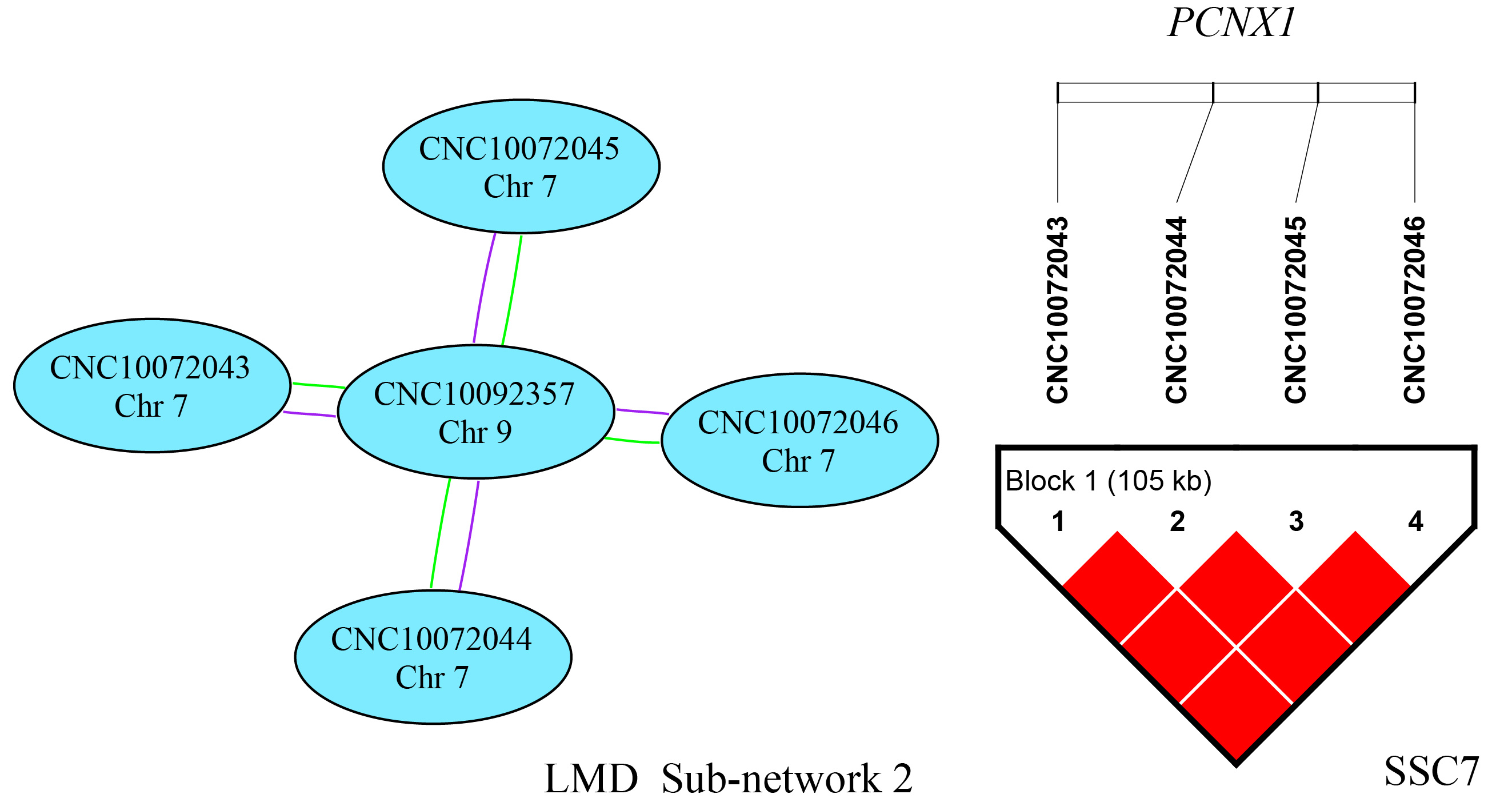

Supplement: Supplementary file 1 [file genes-13-01454-s001.zip › Figure S8. Epistatic sub-network 2 among SNPs affecting LMD and the related LD information.jpg]

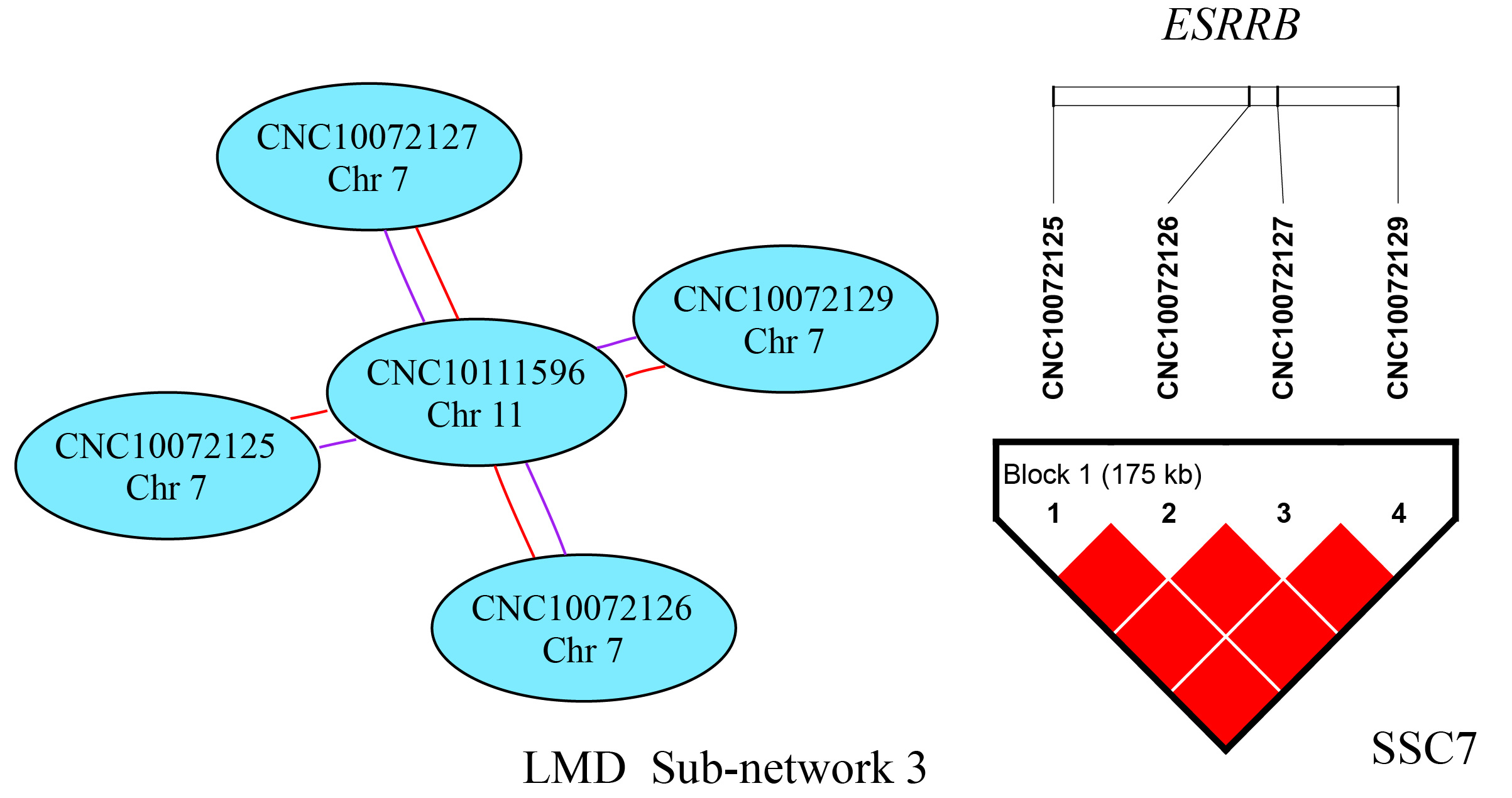

Supplement: Supplementary file 1 [file genes-13-01454-s001.zip › Figure S9. Epistatic sub-network 3 among SNPs affecting LMD and the related LD information.jpg]
